# Characterization of Redox Environment and Tryptophan Catabolism through Kynurenine Pathway in Military Divers’ and Swimmers’ Serum Samples

**DOI:** 10.3390/antiox11071223

**Published:** 2022-06-22

**Authors:** Laura Sánchez Chapul, Gonzalo Pérez de la Cruz, Lucio Antonio Ramos Chávez, Jesús F. Valencia León, Joel Torres Beltrán, Erika Estrada Camarena, Paul Carillo Mora, Daniela Ramírez Ortega, José U. Baños Vázquez, Gabriela Martínez Nava, Alexandra Luna Angulo, Carlos Martínez Canseco, Tiffany Y. Wences Chirino, Juan Ríos Martínez, Verónica Pérez de la Cruz

**Affiliations:** 1 Laboratorio de Enfermedades Neuromusculares, División de Neurociencias Clínicas, Instituto Nacional de Rehabilitación “Luis Guillermo Ibarra Ibarra”, Mexico City 14389, Mexico; abluna@inr.gob.mx (A.L.A.); tiff.wen@ciencias.unam.mx (T.Y.W.C.); 2 Dirección General Adjunta de Sanidad Naval, Secretaría de Marina Armada de México, Mexico City 04830, Mexico; jesus_ferval@hotmail.com; 3 Department of Mathematics, Faculty of Sciences, Universidad Nacional Autónoma de México (UNAM), Mexico City 04510, Mexico; gonzalo.perez@ciencias.unam.mx; 4 Departamento de Neuromorfología Funcional, Dirección de Investigaciones en Neurociencias, Instituto Nacional de Psiquiatría “Ramón de la Fuente”, Mexico City 14370, Mexico; larch_chral@imp.edu.mx; 5 Subdirección de Medicina del Deporte, Instituto Nacional de Rehabilitación “Luis Guillermo Ibarra Ibarra”, Mexico City 14389, Mexico; joetorres@residentes.inr.gob.mx; 6 Laboratorio de Neuropsicofarmacología, Dirección de Investigación, Instituto Nacional de Psiquiatría “Ramón de la Fuente”, Mexico City 14370, Mexico; estrada@imp.edu.mx; 7 División de Neurociencias Clínicas, Instituto Nacional de Rehabilitación “Luis Guillermo Ibarra Ibarra”, Mexico City 14389, Mexico; pcarrillo@inr.gob.mx; 8 Neuroimmunology Laboratory, National Institute of Neurology and Neurosurgery “Manuel Velasco Suárez”, Mexico City 14269, Mexico; drmz@ciencias.unam.mx; 9 Escuela de Búsqueda y Rescate y Buceo, Secretaría de Marina Armada de México, Mexico City 04830, Mexico; juba_0710@hotmail.com; 10 Laboratorio de Gerociencias, Instituto Nacional de Rehabilitación “Luis Guillermo Ibarra Ibarra”, Mexico City 14389, Mexico; gamartinezn@inr.gob.mx; 11 Servicio de Bioquímica, Instituto Nacional de Rehabilitación “Luis Guillermo Ibarra Ibarra”, Mexico City 14389, Mexico; cmartinez@inr.gob.mx; 12 Instituto de Investigación en Ciencias de la Salud de la Secretaria de Marina, Mexico City 04849, Mexico; juan_rios_mtz@yahoo.com.mx; 13 Neurobiochemistry and Behavior Laboratory, National Institute of Neurology and Neurosurgery “Manuel Velasco Suárez”, Mexico City 14269, Mexico

**Keywords:** tryptophan catabolism, kynurenic acid, endurance exercise, redox environment

## Abstract

Endurance and resistance exercises, alone or in combination, induce metabolic changes that affect tryptophan (Trp) catabolism. The kynurenine pathway (KP) is the main route of Trp degradation, and it is modulated by the inflammatory and redox environments. Previous studies have shown that KP metabolites work as myokines that mediate the positive systemic effects related to exercise. However, it is poorly understood how different exercise modalities and intensities impact the KP. The aim of this study was to characterize the effect of two different exercise modalities, military diving and swimming, on the KP and the redox environment. A total of 34 healthy men from the Mexican Navy were included in the study, 20 divers and 14 swimmers, who started and stayed in military training consistently during the six months of the study; 12 Mexican men without fitness training were used as the control group. Physical fitness was determined at the beginning and after 6 months of training; criteria included body composition; serum levels of Trp, kynurenine (KYN), kynurenic acid (KYNA) and 3-hydroxykynurenine (3-HK); the glutathione ratio (GSH/GSSG); and malondialdehyde (MDA).. Results showed a significant loss of body fat in both the diver and swimmer groups. Compared with the control group, divers showed a decrease in Trp and 3-HK levels, but no changes were observed in the KYN/Trp, KYNA/Trp or 3-HK/Trp ratios, while swimmers showed a decrease in KYN levels and an increase in the KYNA and 3-HK levels. Additionally, divers showed a decrease in the GSH/GSSG ratio and an increase in MDA levels, in contrast to the swimmers, who showed a decrease in MDA levels and an increase in GSH/GSSG levels. Our findings suggest a differential shift in the KP and redox environment induced by diving and swimming. Swimming promotes an antioxidant environment and a peripheral overactivation of the KP.

## 1. Introduction

Exercise is widely recognized to improve whole-body performance, and it is one of the most powerful modulators of human metabolism in health and disease [1,2,3]. Aerobic (endurance) exercise has numerous beneficial effects on body composition (BC), but it has also been demonstrated that, in response to endurance exercise (EE), there are regulation processes at the transcriptional level of several metabolic genes that promote increases in muscle energy efficiency, oxidative capacity, resistance to fatigue and cardiovascular function [4,5,6]. In particular, peroxisome proliferator-activated receptor γ co-activator-1α (PGC-1α) is increased by aerobic exercise and promotes phenotypic changes, triggering structural and functional endurance training adaptation, by the induction of a shift towards high-endurance muscle fiber, angiogenesis and mitochondrial biogenesis [7,8,9,10,11,12]. Recently, this transcriptional co-activator was associated with tryptophan (Trp) catabolism through the kynurenine pathway (KP), since PGC-1α1 induces the transcription of kynurenine aminotransferase (KAT) genes in skeletal muscle after exercise in both mice and humans, increasing its product—kynurenic acid (KYNA)—in circulation after exercise [13,14,15]. Moreover, the expression of KATs and the elevated plasma KYNA induced by the exercise were inhibited when PGC-1α was genetically depleted in mouse muscle [14], indicating the strong relation between exercise, PGC-1α1 and Trp catabolism [13].

Trp catabolism through KP produces several metabolites with redox, immunomodulatory and neuroactive properties [16,17,18]. The first step of the KP is Trp oxidation through hepatic tryptophan 2,3-dioxygenase and through extra hepatic indoleamine 2,3-dioxygenase, which lead to L-kynurenine (KYN) production [19]. KYN can be a substrate for three KP enzymes: KATs to produce KYNA, kynureninase to produce anthranilic acid and kynurenine monooxygenase to produce 3-hydroxykynurenine (3-HK) [20]. The intermediate 3-HK produces xanthurenic acid through KATs, or it can be a substrate for kynureninase to produce 3-hydroxyanthranilic acid, which then leads to quinolinic acid (QUIN) production and nicotinamide adenine dinucleotide (NAD+) synthesis de novo through other enzymatic reactions [16]. Fluctuations in KP metabolites have been related to neuropathological conditions, in which common factors, such as oxidative stress and inflammation, are strongly associated with Trp catabolism modulation [21]. In this regard, physical exercise has been shown to have beneficial effects on depression, psychiatric disorders and various neurodegenerative diseases in which KP metabolite alteration has also been reported [22,23]. The initial activation of the KP in response to acute EE is shown by the decrease in Trp and increase in KYN circulating in human serum/plasma. Several studies have shown that Trp catabolism shifts after EE, leading to an increase in KYNA and QUIN plasma levels [24,25,26,27]. These changes in Trp catabolism due to EE are related to a reduction in the KP metabolites that can cross the blood–brain barrier, such as KYN and 3-HK, and prevent an excessive accumulation or a high brain production of KP metabolites that can alter the brain function, such as KYNA [28,29]. However, to date, it is not completely clear how distinct exercise modalities (endurance or resistance), intensities (low, moderate, and high) and duration (chronic or acute) induce a shift in the Trp catabolism and how exercise interplays between the redox environment and inflammation mediators. Herein, we describe the KP metabolism in military scuba divers and surface and helicopter rescue swimmers (SHRS) after chronic endurance and resistance exercise (RE) compared with a sedentary group. This study also provides evidence of the redox environment through the ratio of reduced glutathione (GSH) to oxidized glutathione (GSSG) and lipid peroxidation, as well as neopterin levels as a marker of an inflammatory environment, after 6 months of military training.

## 2. Materials and Methods

### 2.1. Participants

Thirty-four healthy men from the Mexican Navy were included in this study: 20 divers and 14 surface and helicopter rescue swimmers (SHRS) from the School of Search and Rescue and Diving (ESBUSREB, for its acronyms in Spanish) of the Mexican Navy with previously inconsistent exercise. Physical fitness criteria were determined at the beginning (prior to initial physical and tactical training) and end of the training programs (after 6 months), evaluating cardiovascular risk, cardiorespiratory fitness (CRF), metabolic equivalents (METs), as well as BC calculating body fat percentage (BFP) and muscle mass percentage (MMP) by anthropometric measurements. We used a sedentary control group of 12 Mexican men without previous training or exercise records.

This study was approved by the Research and Ethics Committees of the Instituto Nacional de Rehabilitación “Luis Guillermo Ibarra” (CONBIOETICA-09-CEI-03120171207). All naval personnel were informed of the benefits and risks of the investigation prior to signing an institutional informed consent approval document to participation in the study.

### 2.2. Military Training Program

The physical and tactical military training performed was that described in the surface rescue swimmer and diving and underwater works of the ESBUSREB manual of the Mexican Navy. Training was progressive in terms of weight increase, repetitions to successfully completing the sets of each exercise and maximal repetitions. The 6-month training program is described briefly.

*Dry-land training.* This training was the same for divers and rescue swimmers; the training consisted of 20 min sessions two times per week with an intensity of 60–80% of maximum heart rate. This dry-land training consisted of combined anaerobic RE and aerobic EE designed to work on strengthening the upper trunk and core muscles to improve muscular strength and power, as well as neuromuscular development, particularly in the arms and trunk. EE exercises were used to increase CRF. The training consisted of warm-up, resistance force exercises (push-ups, sit-ups, crunch, bench press, bars, kettlebell squats and planks), plyometrics, power exercises (beach sprints and jogging, sledge drag with 45 and 90 pounds of weight and gym work), Fartlek training and functional exercises.

*Tactical divers’ training.* Divers’ physical training program highlighted RE exercises with weight training (necessary to maintain muscle mass and an acceptable fitness level). RE exercises consisted of two-hour immersion at a 1–5-m depth in the pool, 3 times per week, and 1.5-h immersion at a 60-m depth in the sea, 3 to 4 times per week, over 6 months whilst breathing compressed air (21% oxygen and 79% nitrogen) in an open circuit system and wearing neoprene suits (80-foot double scuba, visor, fins, hood, octopus’s booties, watch and leaded ballast belts) in sea diving. This training included apneas at a low depth and horizontal apneas of 25 m to learn to control breathing, stay calm and purge the viewfinder under water. The diving activities consisted of rescue diving, revision of axles, propellers, discharges and suctions of ships.

*Tactical surface and helicopter rescue swimmers’ training.* SHRS were equipped with the mental capacity, swimming technique, efficiency, flexibility, strength and endurance to work for 30 min in heavy seas. Tactical training consisted of EE conditioning through crawl swimming with equipment (800 m/14 min, 1500 m/28 min and 2000 m/42 min), and body towing (800 m/28 min) to learn how to approach, carry and release survivors; 4 apneas, which consisted of 25 m in length at 40 s intervals, were induced 3 times per week.

### 2.3. Physical Fitness Criteria

*Cardiovascular risk.* The stratification of the cardiovascular risk was determined before starting the military training program (beginning) through a clinical evaluation, including spirometry and electrocardiogram at rest.

*Cardiorespiratory fitness.* CRF was evaluated using the Course–Navette test, which measures maximal aerobic power and indirectly the maximum oxygen uptake (METs) [30]. This test was performed at the beginning and at the end of training program, in groups of eight participants on the same day and the same running track to ensure precision and to accomplish standardization among the participants.

*Body Composition.* BC was assessed at the beginning and at the end of training program by anthropometric measurements through three indices: (a) body mass index (BMI); (b) BFP and MMP, and (c) waist circumference (WC). The weight, height, diameters, circumference and 25-skinfolds were obtained according to the International Society for the Advancement of Kinanthropometry (ISAK) guidelines [31]. To avoid technical measurement errors, all measures were carried out, on the same day, in the same session.

*Anthropometric measurements.* Prior to the anthropometric measurements, naval personnel were asked not to have carried out any type of exercise and to undergo a 12-h fasting, wearing only shorts without lotions, oils or body creams. A calibrated skinfold caliper (Slim Guide) and a short-branch anthropometer (Ces-corf) were used to measure skinfold thicknesses. The measurements were made by two certified level 2 anthropometrists.

*BMI.* This index was calculated by dividing weight (in kg) by height (in square meters) (kg/m^2^). Height and weight were obtained using a portable stadiometer (Seca Model 213, Hamburg, Germany) and a calibrated digital flat scale (Omron Brand model Hbf-514 C), respectively.

*Muscle mass and body fat percentage.* MMP and BFP were calculated using the Drinkwater and Ross equation [32,33] and the Siri formula from the 3-fold Jackson and Pollock formula for body density, respectively [34]. The reference interval used for MMP was that reported for men who practice triathlons, with a 46.7 to 51–55% of muscle mass [35]. The reference intervals for BFP were those established by the American College of Sports Medicine, whose values are 7.9–10.5% (18–29 years), 12.4–14.9% (30–39 years), 15–17.5% (40–49 years) [36] and BFP ≤ 25% for male naval personnel.

*WC.* WC was the screening tool used to estimate weight status in relation to potential disease risk. This tool was measured using a measuring tape (Rosscrafty Lufkin) according to the ISAK protocol for the diagnosis of metabolic diseases in men. A WC ≤ 90 cm is considered as healthy while > 90 cm as a risk [37].

### 2.4. Materials

3-HK, KYN, KYNA, Trp, glutathione reduced form (GSH), oxidized glutathione (GSSG), N-ethylmaleimide (NEM), O-phthaldialdehyde (OPA), thiobarbituric acid (TBA) and trichloroacetic acid (TCA) were obtained from Sigma-Aldrich (St. Louis, MO, USA). All other chemicals were of the highest commercially available purity. Solutions were prepared using deionized water.

#### 2.4.1. Determination of Redox Status and KP Metabolites in Serum

Blood samples. Blood samples were collected to determine the serum levels of malondialdehyde (MDA, a final product of lipoperoxidation), glutathione levels and KP metabolites. Two peripheral blood samples were taken at different times from each participant. The first one was taken prior to starting a military training program. The second one was taken 6 months later once the training was finished. For both samples (beginning and 6 moths), the participants were asked not to have carried out any type of exercise and to undergo a 12-h fasting. Blood samples were collected by venipuncture, in a BD vacutainer serum tube (Vacutainer, Becton, Dickinson and Company, Franklin Lakes, NJ, USA); serum was immediately separated by centrifugation, then samples were passed through a 0.2-µm filter. Later, samples were separated in two tubes: (1) the first one for immediate determination of the redox state (GSH/GSSH ratio and MDA) and (2) the second was stored at −80 °C until analysis for kynurenines determination.

#### 2.4.2. Serum GSH and GSSG Determination

An adapted fluorometric method was used to evaluate GSH and GSSG [38,39]. In brief, serum samples (50 µL) were treated with metaphosphoric acid (150 µL at 5% (*w*/*v*)), mixed immediately and placed on ice to confer stability for GSH quantification. Then, samples were centrifuged at 14,000× *g* for 20 min at 4 °C. The supernatant was used for GSH (5 µL) and for GSSG (30 µL) determination. For GSSG determination, NEM and dithionite were added, then the samples were treated with OPA to obtain the isoindole. Fluorescence was determined at 370 nm of excitation and 420 nm of emission (FLx800 Multimode Lector BioTex, Houston, TX, USA). The concentrations of GSH and GSSG were obtained by interpolation in the standard curve. The results are expressed as µmol/L.

#### 2.4.3. Lipid Peroxidation

Lipoperoxidation was evaluated through the production of thiobarbituric acid reactive species (TBA-RS) [40]. Serum samples (125 µL) were added with 250 µL of TBA solution (0.375 g of TBA + 15 g of TCA + 2.54 mL of HCl in 100 mL) and boiled for 15 min. Then, the samples were placed on ice and centrifuged at 9800× *g* for 10 min. Later, the optical density of the supernatant was determined at a wavelength of 532 nm. Results were expressed as a micromolar of MDA (mol/L).

#### 2.4.4. Kynurenines Determination

Serum samples were treated with 200 µL of perchloric acid (6%) and vigorously mixed. Then samples were centrifuged at 14,000× *g* and 4 °C. The supernatant was used for kynurenines determination [41,42,43,44]. Trp was measured by a reverse HPLC method with fluorescence detection using ZORBAX Eclipse AAA column (3.5-μm, 4.6 × 150 mm, Agilent, Santa Clara, CA, USA), and isocratic elution with a mobile phase (pH 4.2) containing 100 mM of zinc acetate and 3% of acetonitrile at a flow rate of 1 mL/min. Trp was detected at excitation wavelength: 254 nm and emission wavelength: 404 nm using a Perkin Elmer fluorescence detector (model S200a). The retention time of Trp was ~5 min. KYNA quantification used an Eclipse XDB-C18 reverse phase column (5-μm, 4.6 × 150 mm, Agilent, Santa Clara, CA, USA) and a mobile phase consisting of 50 mM sodium acetate, 250 mM of zinc acetate and 3% of acetonitrile (pH 6.2), at a flow rate of 1 mL/min. KYNA was determined at an excitation wavelength of 344 nm and an emission wavelength of 398 nm. The retention time for KYNA was ~7 min. For KYN determination the same KYNA conditions were used but without acetonitrile in the mobile phase. KYN was detected at an excitation wavelength of 368 nm and an emission wavelength of 480 nm with a retention time ~10 min.

The 3-HK levels were measured using a reverse HPLC method with electrochemical detection. The sample (40 μL) was injected onto an Adsorbosphere Catecholamine C18 reverse phase column (3-μm, 4.6 mm × 100 mm, Fisher Scientific, Hampton, NH, USA) at a constant flow rate of 0.6 mL/min with a mobile phase containing 9% triethylamine, 0.59% phosphoric acid, 0.27 mM EDTA and 8.9 mM heptane sulfonic acid. The 3-HK retention time was ~11 min.

#### 2.4.5. Serum Neopterin

Serum neopterin was measured using a commercially available competitive ELISA kit (Wuhan Fine Biotech Co., Ltd., Wuhan, China) according to the manufacturer’s protocol. This immunoassay had an intra- and inter-assay coefficient of variance <8% and <10%, respectively, and a sensitivity of 0.094 ng/mL.

### 2.5. Statistical Analysis

The changes in BC, physical fitness, Trp catabolism and neopterin levels at the beginning and end of training were assessed using the Wilcoxon signed-rank test. Comparisons between groups were performed using the Kruskal–Wallis test with Dunn’s test for multiple pairwise comparisons. Spearman correlation was used to assess the association between variables. Statistical significance was set at *p* < 0.05. All statistics were calculated with Graph Prism 9.1.0. (GraphPad, San Diego, CA, USA).

## 3. Results

### 3.1. Description of the Study Population

A total of 46 males were included in this study. The mean age of the control group (sedentary individuals) was 23.1 ± 0.66, of the diver group was 27.2 ± 0.86 and of the SHRS group was 28.6 ± 1.14 years (Table 1). The three groups in this study were similar in height and did not present cardiometabolic risk (WC ≤ 90 cm).

### 3.2. BC, Trp Catabolism and Redox Environment Parameters at the Beginning up to 6 Months after the Military Training Program in Divers’ and SHRS’ Serum

**Figure 1:**
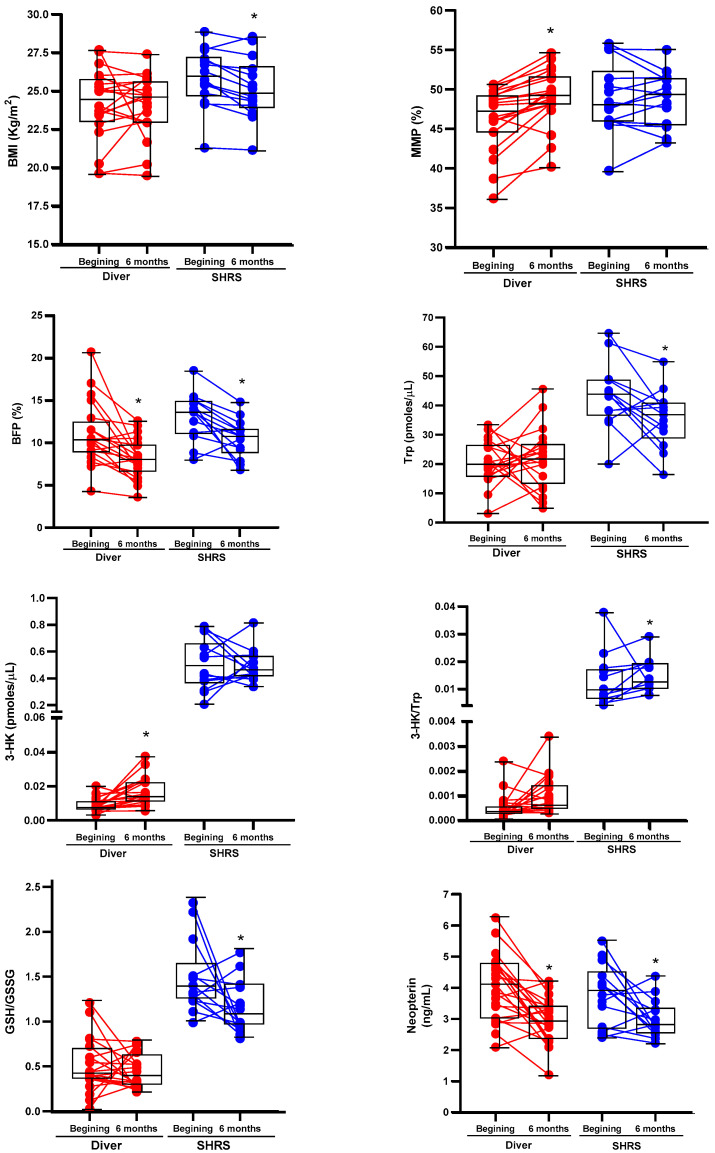
Boxplots associated with significant changes of BC, Trp catabolism and redox environment of divers and SHRSs after six of the military training program. The graphs also include the values at the beginning and at the end of program for each individual. * *p* < 0.05 vs. beginning, based on Wilcoxon signed-rank test.

The changes in the BC, physical fitness, Trp catabolism and redox environment in divers and SHRSs at the beginning and end of the military training program are shown in Table 2 and Table 3, respectively. Figure 1 shows the boxplots associated with those variables with significant changes. After six months of training, divers maintained an average BMI in the normal weight category (<24.9 kg/m^2^), with an improved BC by decreasing BFP and increasing MMP, without changes in METs or redox markers. Additionally, this group showed an increase in 3-HK serum levels and a decrease in neopterin levels (an inflammation marker) (Table 2). The SHRS group lost weight, maintained a BMI in the overweight category (˃25 kg/m^2^) despite decreasing BFP and improved CRF (METs ˃ 12). Additionally, this group showed a decrease in Trp serum levels, neopterin and the GSH/GSSG ratio, and an increase in the Trp/3-HK ratio (Table 3).

### 3.3. Changes in Trp Catabolism and Redox Environment Induced by the Type of Exercise

**Figure 2:**
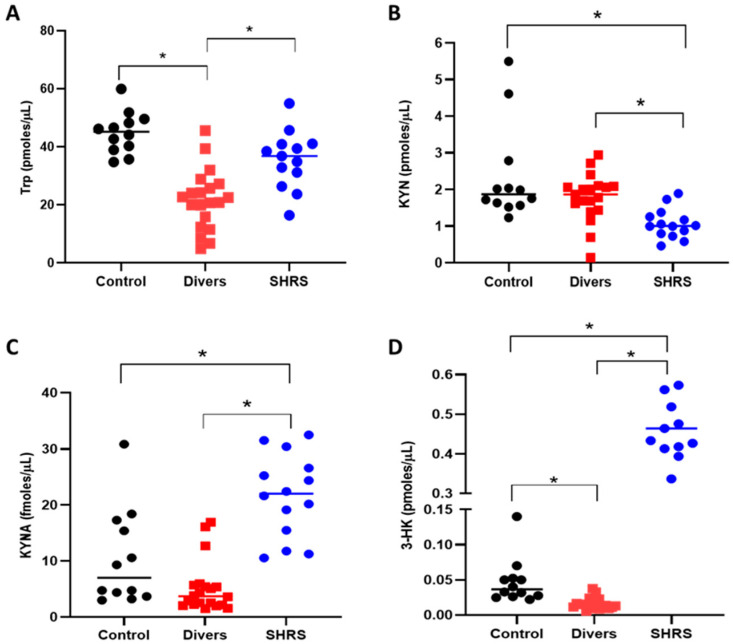
Effect of different types of exercise on serum KP metabolites: control (*n* = 12), diver (*n* = 20) and SHRS (*n* = 14) groups. (**A**) Trp serum levels, (**B**) KYN serum levels, (**C**) KYNA serum levels and (**D**) 3-HK serum levels. Data are represented by the median. * *p* < 0.05 based on the Kruskal–Wallis test with Dunn’s test for multiple pairwise comparisons.

The serum KP metabolites and redox environment induced in the diver and SHRS groups at the end of training (6 months) were compared to the serum levels of the control group (Figure 2). The diver group showed a significant decrease (around 52%) in Trp levels compared to the control group (Figure 2A), while the SHRS group did not show differences in Trp when compared to the control group. However, contrary to our expectations, the levels of KYN and KYNA did not change in the diver group (Figure 2B,C), and only the 3-HK serum levels decreased, by 35%, compared to the control group (Figure 2D). In the case of the SHRS group, the serum KYN levels decreased by 55% compared to the control group, while serum KYNA and 3-HK levels increased by 2-fold and 10-fold, respectively, compared to the control group, suggesting an overactivation of KP (Figure 2C,D).

**Figure 3:**
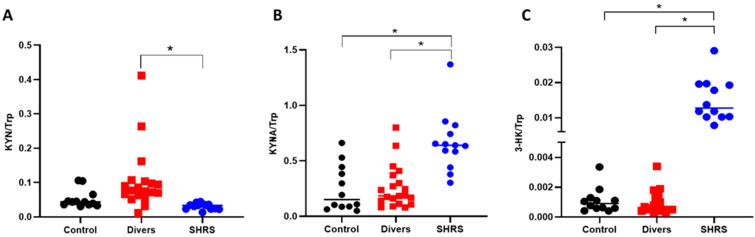
Effect of different types of exercise on serum KP ratios: control (*n* = 12), diver (*n* = 20) and SHRS (*n* = 14) groups. (**A**) KYN/Trp ratio, (**B**) KYNA/Trp ratio and (**C**) 3-HK/Trp ratio. Data are represented by the median. * *p* < 0.05 based on the Kruskal–Wallis test with Dunn’s test for multiple pairwise comparisons.

When the ratios were calculated, the interpretation was similar to that obtained with the serum metabolites levels, since the diver group did not show a difference compared to the control group. The SHRS group maintained a higher ratio of KYNA/Trp and 3-HK/Trp, confirming the over activation of KP in this group (Figure 3).

**Figure 4:**
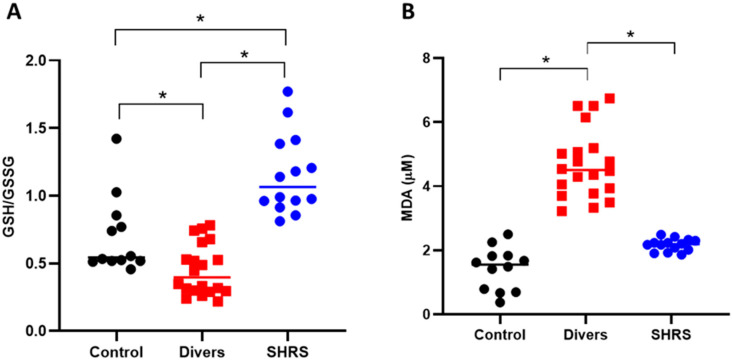
Effect of different types of exercise on serum redox environment: control (*n* = 12), diver (*n* = 20) and SHRS (*n* = 14) groups. (**A**) GSH/GSSG ratio and (**B**) MDA serum levels. Data are represented by the median. * *p* < 0.05 based on the Kruskal–Wallis test with Dunn’s test for multiple pairwise comparisons.

The redox environment was evaluated through the levels of the main antioxidant (GSH) and MDA (Figure 4). The diver group showed an oxidant environment since the GSH/GSSG ratio decreased by 36% compared to control, and the MDA levels increased by 3-fold vs. the control. The SHRS group was completely different, since the GSH/GSSG ratio increased by 65% compared to the control group, and no differences were found in MDA levels compared to the control group.

**Figure 5:**
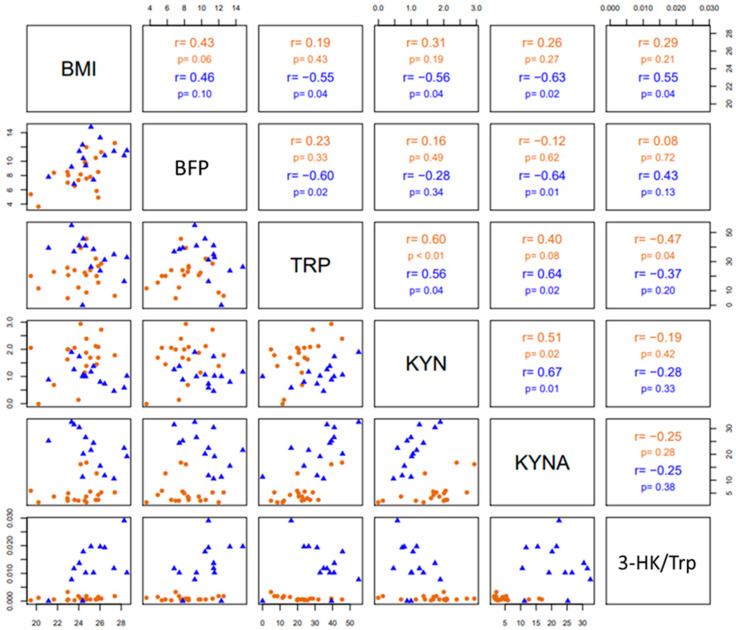
The lower triangular matrix contains the scatterplot for each pair of variables for divers and SHRSs. The upper triangular matrix includes the Spearman’s rank correlation coefficient (r) with its respective associated *p*-value (*p*) calculated for each group.

Pairwise correlations among BMI, BFP, Trp, KYN, KYNA and the 3-HK/Trp ratio are shown in Figure 5. In both groups (divers and SHRS), Trp levels (pmoles/µL) correlated positively with KYN levels (pmoles/µL), as well as KYN with KYNA (fmoles/µL) circulating levels. In SHRS, Trp levels correlated positively with KYNA levels, and BMI with the 3-HK/Trp ratio; additionally, there was a negative correlation of BMI with KYNA, KYN and Trp levels, as well as of BFP with KYNA and Trp levels. Finally, Trp levels correlated negatively with the 3-HK/Trp ratio in divers.

## 4. Discussion

In this study, we explored the KP metabolites and redox environment in the serum of Mexican naval personnel to investigate the changes associated with swimming and diving, in combination with a chronic EE and RE as part of a military training program. Our results showed the differences in the Trp catabolism and redox environment of divers and SHRSs over the 6 months of training. After six months of consistent exercise, both groups showed improved BC by losing body fat, but only divers showed increased MMP. The SHRS group lost weight, and despite decreasing BMI, they remained in the overweight category, but with good BC, and showed improved cardiorespiratory fitness (METs ˃ 12); additionally, this group showed decreased circulating Trp levels and an increased 3-HK/Trp ratio, suggesting an overactivation of the KP, leading to de novo NAD+ production. In addition, when the glutathione ratio was determined in both groups after six months of training, only the SHRS group showed increased levels of this antioxidant compared with the levels observed at the beginning of training, while neopterin was decreased.

The negative correlation of BMI with KYNA, KYN and Trp levels, as well as of BFP with KYNA and Trp, and the positive correlation of BMI with the 3-HK/Trp ratio in SHRSs suggest that improving SHRSs’ BC by losing body fat but maintaining adequate muscle mass could influence Trp catabolism throughout the increment in free Trp in the blood due to lipolysis during exercise, leading to an increase in free fatty acids, which bind to albumin, releasing Trp into the blood [15]. It could also influence the anti-inflammatory response due to the chronic exercise intervention (>12 weeks), which may lead to reductions in IDO activity as a result of visceral fat mass loss, the major source of low-grade inflammation and meta-inflammation [45,46].

To evidence the differential metabolism of Trp, the redox environment and the long-term adaptation effect between diving and swimming, we decided to add a sedentary control group. The results of our study showed that despite having the same average EE and RE in the dry-land training, the metabolism shifted between divers and swimmers. The diver group presented reduced Trp and 3-HK circulating levels when compared to the control group; however, there were no changes in KYN/Trp, KYNA/Trp or 3-HK/Trp ratios, indicating that the Trp catabolism through KP is not modified by diving after 6 months of training. In addition, the diver group presented an oxidant environment when compared to the control group since the GSH/GSSG ratio decreased and the MDA levels increased (Figure 4). These circulation changes in the diver group could be associated with the physiological stress that the underwater environment imposes since during diving, the divers increased their breathing resistance due to their exposure to compressed air and hyperoxia [47,48], in addition to the elevated physical demands due to the weight of the diving equipment and the oxygen costs to overcome resistance to water, underwater pressure and the formation of intravascular nitrogen bubbles [49,50,51]. As MDA levels seem to be influenced by diving depths, producing a significant increase in serum MDA 3 h after diving at a 50-m depth [52,53], the oxidizing response observed in our diving group is due to the chronic immersion (1.5-h immersion at a 60-m depth in the sea, 3 to 4 times per week for 6 months), which led to the production of ROS, a condition that, together with the decrease in GSH, shifts the redox balance in favor of long-term oxidative stress.

Interestingly, unlike the diver group, the SHRS group showed an increase in Trp catabolism, confirmed by the decrease in KYN circulating levels, as well as by the increment in KYNA and 3-HK circulating levels, and confirmed by the increase in KYNA/Trp and 3-HK/Trp ratios vs. the control group. The increase in circulating KP metabolites could be associated with the higher oxygen consumption and the requirement of energy during SHRSs’ tactical training, in which EE predomination led to an overactivation of KP, providing the cofactor NAD+ de novo to be used in the energetic metabolism activated by the EE [12,54,55]. Moreover, the evidence shown in our study is consistent with recent studies, which demonstrated an increase in circulating KYNA following EE [15,56,57]. As mentioned previously, this shift in KYNA serum levels is related to the stimulation of both mRNA [13,15] and the protein expression of KATs in muscle [14,58] through PGC-1α1 [14,59]. Interestingly, the SHRS group maintained a redox balance in favor of longer-lasting antioxidant capacity to counter oxidative stress, probably due to the activated aerobic metabolism of the regular and continuous bouts of EE [60,61,62,63,64] and the antioxidant and scavenger properties of the circulating levels of KYNA and 3-HK [65,66,67,68]. Additionally, a recent report suggests that KP metabolism supports the aspartate biosynthesis and mitochondrial function of the trained muscle, increasing the energy efficiency of glucose oxidation, and this mechanism is dependent on PGC-1α1 [13].

Shifts in KP metabolism induced after a 12-week exercise program (two resistance training sessions and one high-intensity interval training session) have been observed in healthy older men over 65 years of age. In this case, all the isoforms of KAT increased in skeletal muscle in healthy older men, suggesting exercise as a potential KP modulator for aging to improve the declines in mood and cognition induced by age [14,58]. Another study showed that high-intensity interval training increased the KYNA/QUIN ratio and reduced impulsivity in emotionally impulsive humans [69]. However, the study of the impact of diverse modalities or the intensities of exercise and their influence on KP is complex, even when it is well described that exercise induces a co-activator that promotes KAT expression in muscle, leading to an increase in circulating KYNA levels, which prevents its accumulation or production in the brain. Moreover, the Trp catabolism can be modulated by several environmental conditions, such as stress, inflammation, medication and diet, and these factors should be considered in future studies. In this context, a recent study observed that after a 12-week training period at three different intensities, the patients affected by mild-to moderate depression experienced positive effects on their mood and cardiovascular fitness, but showed no difference in plasma KYN and KYNA, suggesting that, in this case, exercise did not lead to long-lasting changes in plasma KP metabolites [70]. Nevertheless, it has been described that KAT muscle induction through exercise shifts the KYN metabolism towards the enhanced synthesis of KYNA, giving protection from stress-induced depression in PGC-1α1 skeletal muscle-specific transgenic mice [14]. The challenge for future research is to determine whether these changes in Trp catabolism induced by exercise are related to an improvement in the mood or cognitive functions of healthy subjects and patients with pathologies that involve fluctuations in KP metabolites, whilst considering the possible confounding factors, as a pharmacological intervention.

Our study has several limitations. First, the sample size was small in both study groups. Second, no major changes were observed in the 6-month evaluation period since both groups had practiced swimming and diving once before entering the ESBUSREB, allowing their bodies to adapt to their respective modality of exercise. Third, we had no control over the diets of the study groups; therefore, we do not know the content and the availability of the macronutrients that may have influenced Trp catabolism. Fourth, the degree of stress, depression and anxiety was not evaluated in this population; however, we are considering these evaluations together with CRF—which has been related to improve memory performance and as-sociated with lower depressive symptoms [71,72] in future work involving naval personnel, given the potential of neurological functions of Trp metabolites and exercise for mental health. Finally, the lipid peroxidation was estimated in serum samples by TBA-RS, which measures the MDA concentration; however, other compounds could cause overestimation of MDA concentration, for which in futures studies the lipoperoxidation will be confirmed by chromatographic separation of MDA–TBA adduct [40].

## 5. Conclusions

Herein, the Trp catabolism and redox status adaptation induced by two exercise modalities, swimming and diving, was characterized. Swimming induced a shift in circulating Trp metabolites and improved the antioxidant environment compared to a sedentary population. These findings pave the way for new studies in which swimming at different intensities could be studied as a potential therapeutic adjuvant in many diseases presenting fluctuations in brain KP metabolites with neuroactive properties.

## Figures and Tables

**Table 1:** Characteristics of subjects included in the study. Data are mean ± SEM.

	Controls	Divers	SHRS
	(*n* = 12)	(*n* = 20)	(*n* = 14)
**Age**	23.1 ± 0.66	27.2 ± 0.86	28.6 ± 1.14
**Heigh (m)**	1.75 ± 0.02	1.74 ± 0.01	1.69 ± 0.02
**WC (cm)**	≤90	≤90	≤90

**Table 2:** Descriptive statistics of divers’ characteristics at the beginning and after 6 months of military training program.

		Min	Percentile 25	Median	Percentile 75	Max	Mean	*p* Values Wilcoxon Signed-Rank Test
**Weight** **(kg)**	Beginning	60.10	66.98	74.10	76.33	83.80	72.76	0.9345
	6 months	62.60	69.78	72.35	77.65	82.80	72.88	
**BMI** **(kg/m^2^)**	Beginning	19.62	23.02	24.49	25.82	27.68	24.13	0.8771
	6 months	19.48	22.98	24.65	25.67	27.41	24.12	
**MMP (%)**	Beginning	36.20	44.60	47.25	49.25	50.60	46.27	0.0001 *
	6 months	40.20	48.10	49.25	51.63	54.60	49.07	
**BFP (%)**	Beginning	4.30	8.925	10.40	12.55	20.70	11.02	0.0005 *
	6 months	3.60	6.625	8.10	9.85	12.60	8.18	
**METs**	Beginning	9.07	10.30	11.14	12.48	14.16	11.30	0.7680
	6 months	8.10	9.935	11.67	12.53	14.16	11.26	
**Trp** **(pmoles/µL)**	Beginning	3.06	15.63	19.86	26.50	33.40	20.66	0.7285
	6 months	4.87	13.19	21.65	26.83	45.58	21.66	
**KYN** **(pmoles/µL)**	Beginning	1.26	1.58	1.88	2.56	3.20	2.03	0.5791
	6 months	0.14	1.65	2.00	2.09	2.94	1.84	
**KYNA** **(fmoles/µL)**	Beginning	2.82	3.50	5.29	5.88	8.84	5.02	0.3488
	6 months	1.51	2.21	3.72	5.63	16.93	5.23	
**3-HK** **(pmoles/µL)**	Beginning	0.003	0.006	0.008	0.011	0.02	0.01	0.0001 *
	6 months	0.006	0.011	0.014	0.023	0.038	0.02	
**KYN/Trp**	Beginning	0.054	0.081	0.087	0.153	0.629	0.143	0.3778
	6 months	0.012	0.064	0.077	0.100	0.412	0.104	
**KYNA/Trp**	Beginning	0.095	0.171	0.203	0.325	0.672	0.262	0.8596
	6 months	0.080	0.113	0.187	0.371	0.800	0.265	
**3-HK/Trp**	Beginning	0.0001	0.0003	0.0004	0.0006	0.0024	0.0006	0.0689
	6 months	0.0003	0.0005	0.0007	0.0015	0.0034	0.0010	
**MDA** **(µM)**	Beginning	3.22	3.79	4.48	4.94	5.61	4.41	0.3162
	6 months	3.22	3.82	4.51	5.16	6.74	4.69	
**GSH/GSSG**	Beginning	0.03	0.36	0.42	0.70	1.21	0.51	0.4749
	6 months	0.22	0.30	0.40	0.63	0.78	0.45	
**Neopterin** **(ng/mL)**	Beginning	2.09	3.023	4.11	4.79	6.25	4.04	0.0002 *
	6 months	1.203	2.374	2.95	3.43	4.21	2.99	

BMI: body mass index; MMP: muscle mass percentage; BFP: body fat percentage; METs: metabolic equivalents; Trp: tryptophan; KYN: kynurenine; KYNA: kynurenic acid; 3-HK: 3-hydroxykynurenine; MDA: malondialdehyde; GSH/GSSG: glutathione ratio. * *p* < 0.05 vs. Beginning.

**Table 3:** Descriptive statistics of SHRSs’ characteristics at the beginning and after 6 months of military training program.

		Min	Percentile 25	Median	Percentile 75	Max	Mean	*p* Values Wilcoxon Signed-Rank Test
**Weight** **(kg)**	Beginning	60.80	68.40	72.25	77.38	92.50	73.89	0.0132 *
	6 months	60.40	66.03	69.95	77.15	90.60	72.18	
**BMI** **(kg/m^2^)**	Beginning	21.29	24.69	26.00	27.25	28.87	25.90	0.0031 *
	6 months	21.15	23.93	24.90	26.66	28.54	25.19	
**MMP (%)**	Beginning	39.70	45.98	48.10	52.33	55.80	48.89	0.8672
	6 months	43.30	45.53	49.40	51.43	55.00	48.89	
**BFP (%)**	Beginning	8.00	11.10	13.65	15.00	18.50	13.19	0.0023 *
	6 months	6.80	8.85	10.80	11.70	14.80	10.52	
**METs**	Beginning	8.86	10.24	11.53	12.52	13.23	11.32	0.0067 *
	6 months	8.01	11.77	12.63	13.43	14.53	12.42	
**Trp** **(pmoles/µL)**	Beginning	19.95	36.50	43.84	48.76	64.69	43.52	0.0479 *
	6 months	16.36	28.74	36.82	40.93	54.90	35.90	
**KYN** **(pmoles/µL)**	Beginning	0.41	0.89	1.06	1.37	2.05	1.13	0.8077
	6 months	0.46	0.78	1.01	1.29	1.89	1.07	
**KYNA** **(fmoles/µL)**	Beginning	6.61	13.00	17.10	21.29	57.26	19.64	0.1726
	6 months	10.57	14.57	22.01	27.54	32.52	21.65	
**3-HK** **(pmoles/µL)**	Beginning	0.206	0.345	0.435	0.693	0.787	0.500	0.9990
	6 months	0.337	0.416	0.454	0.568	0.814	0.495	
**KYN/Trp**	Beginning	0.011	0.186	0.023	0.038	0.058	0.028	0.4973
	6 months	0.013	0.023	0.034	0.036	0.045	0.031	
**KYNA/Trp**	Beginning	0.153	0.291	0.419	0.727	0.885	0.478	0.1099
	6 months	0.303	0.513	0.641	0.782	1.370	0.667	
**3-HK/Trp**	Beginning	0.0042	0.0066	0.0099	0.0173	0.0379	0.0133	0.0342 *
	6 months	0.0078	0.0102	0.0128	0.0195	0.0291	0.0151	
**MDA** **(µM)**	Beginning	1.81	2.19	2.34	2.42	3.47	2.36	0.0652
	6 months	1.86	1.99	2.19	2.31	2.49	**2.17**	
**GSH/GSSG**	Beginning	0.99	1.23	1.36	1.61	2.32	**1.49**	0.0494 *
	6 months	0.81	0.95	1.06	1.39	1.77	1.16	
**Neopterin** **(ng/mL)**	Beginning	2.41	2.70	3.91	4.52	5.51	3.79	0.0353 *
	6 months	2.22	2.55	2.83	3.36	4.37	3.00	

BMI: body mass index; MMP: muscle mass percentage; BFP: body fat percentage; METs: metabolic equivalents; Trp: tryptophan; KYN: kynurenine; KYNA: kynurenic acid; 3-HK: 3-hydroxykynurenine; MDA: muscle mass percentage; GSH/GSSG: glutathione ratio. * *p* < 0.05 vs. Beginning.

## Data Availability

Data is contained within the article.
